# The Cys-Arg/N-End Rule Pathway Is a General Sensor of Abiotic Stress in Flowering Plants

**DOI:** 10.1016/j.cub.2017.09.006

**Published:** 2017-10-23

**Authors:** Jorge Vicente, Guillermina M. Mendiondo, Mahsa Movahedi, Marta Peirats-Llobet, Yu-ting Juan, Yu-yen Shen, Charlene Dambire, Katherine Smart, Pedro L. Rodriguez, Yee-yung Charng, Julie E. Gray, Michael J. Holdsworth

**Affiliations:** 1 School of Biosciences, University of Nottingham, Loughborough LE12 5RD, UK; 2 Department of Molecular Biology and Biotechnology, University of Sheffield, Sheffield S10 2TN, UK; 3 Instituto de Biología Molecular y Celular de Plantas, Consejo Superior de Investigaciones Científicas-Universidad Politécnica de Valencia, Ciudad Politécnica de la Innovación, 46022 Valencia, Spain; 4 Agricultural Biotechnology Research Center, Academia Sinica, 128 Academia Road Section 2, Taipei, Taiwan 11529, ROC; 5 SABMiller Limited, ABInBev House, Church Street West, Woking, Surrey GU21 6HT, UK

**Keywords:** N-end rule pathway, abiotic stress response, ERFVII transcription factors, proteolysis6, BRAHMA, nitrate reductase, nitric oxide

## Abstract

Abiotic stresses impact negatively on plant growth, profoundly affecting yield and quality of crops. Although much is known about plant responses, very little is understood at the molecular level about the initial sensing of environmental stress. In plants, hypoxia (low oxygen, which occurs during flooding) is directly sensed by the Cys-Arg/N-end rule pathway of ubiquitin-mediated proteolysis, through oxygen-dependent degradation of group VII Ethylene Response Factor transcription factors (ERFVIIs) via amino-terminal (Nt-) cysteine [1, 2]. Using *Arabidopsis* (*Arabidopsis thaliana*) and barley (*Hordeum vulgare*), we show that the pathway regulates plant responses to multiple abiotic stresses. In *Arabidopsis*, genetic analyses revealed that response to these stresses is controlled by N-end rule regulation of *ERFVII* function. Oxygen sensing via the Cys-Arg/N-end rule in higher eukaryotes is linked through a single mechanism to nitric oxide (NO) sensing [3, 4]. In plants, the major mechanism of NO synthesis is via NITRATE REDUCTASE (NR), an enzyme of nitrogen assimilation [5]. Here, we identify a negative relationship between NR activity and NO levels and stabilization of an artificial Nt-Cys substrate and *ERFVII* function in response to environmental changes. Furthermore, we show that *ERFVII*s enhance abiotic stress responses via physical and genetic interactions with the chromatin-remodeling ATPase BRAHMA. We propose that plants sense multiple abiotic stresses through the Cys-Arg/N-end rule pathway either directly (via oxygen sensing) or indirectly (via NO sensing downstream of NR activity). This single mechanism can therefore integrate environment and response to enhance plant survival.

## Highlights

• The Cys-Arg/N-end rule pathway controls responses to multiple abiotic stresses
• NITRATE REDUCTASE enhances N-end rule, and opposes ERFVII substrate, action
• ERFVII substrates interact with chromatin remodeling ATPase BRAHMA

## Graphical Abstract

**Figure:**
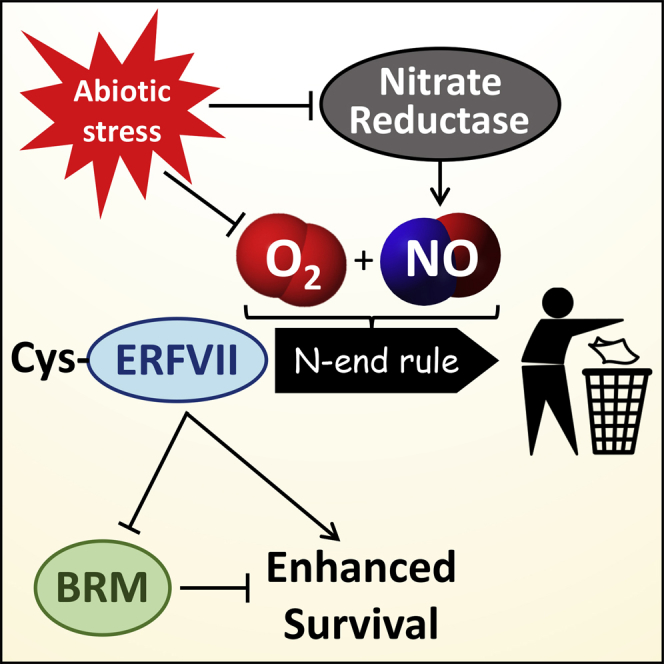

## Teaser

Vicente et al. show that the Cys-Arg/N-end rule pathway is a general sensor of abiotic stress. N-end rule action on ERFVII transcription factor substrates controls stress tolerance, influenced by NITRATE REDUCTASE and interactions with chromatin remodeling ATPase BRAHMA. This mechanism integrates environment and response to enhance survival.

## Results and Discussion

### The Cys-Arg/N-End Rule Pathway Controls ERFVII-Mediated Tolerance to Multiple Abiotic Stresses

**Figure 1:**
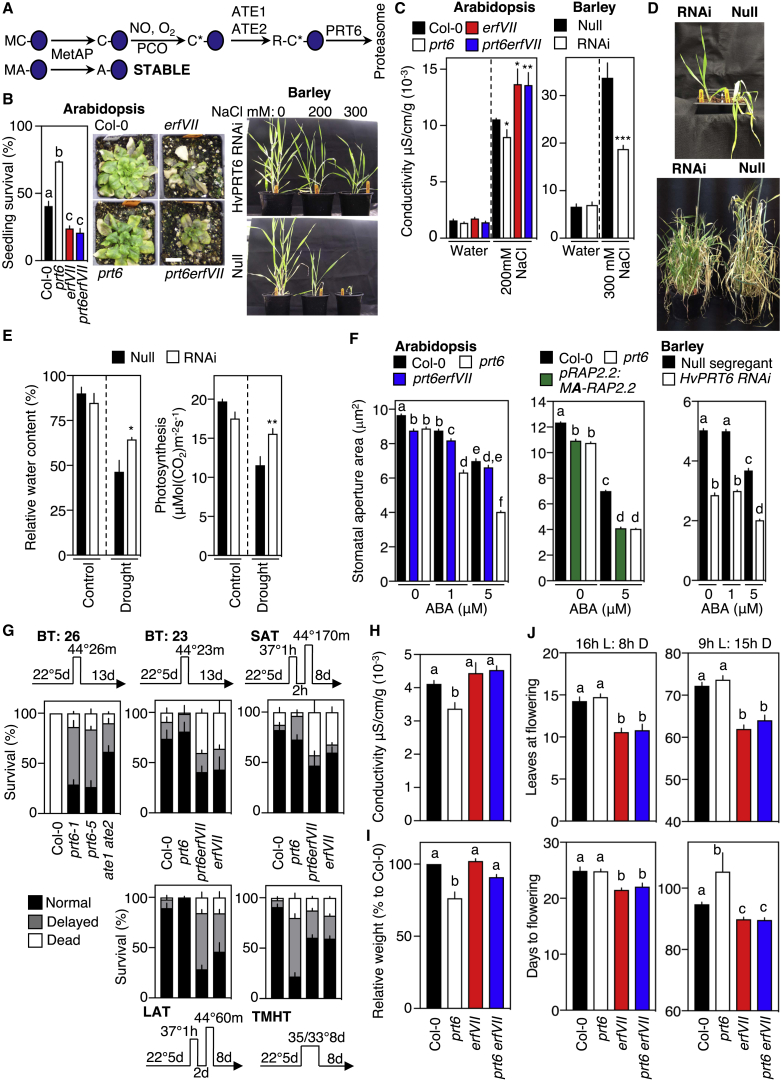
The N-End Rule Pathway Influences Tolerance to Multiple Abiotic Stresses (A) Diagrammatic representation of the Cys-Arg/N-end rule pathway. ATE, ARGINYL TRANSFERASE; MetAP, MET AMINO PEPTIDASE; PCO, PLANT CYSTEINE OXIDASE; PRT, PROTEOLYSIS. C^∗^ indicates oxidized Cys. Single letter amino-acid codes are used; blue ovals are proteins. (B) Survival of 3-day-old *Arabidopsis* seedlings transferred to half strength Murashige and Skoog media (1/2MS) media containing 200 mM NaCl for 7 days followed by 5 days recovery on 1/2MS. Images are of mature *Arabidopsis* and barley plants in soil watered with salt. The *Arabidopsis* scale bar represents 1 cm. (C) Conductivity (electrolyte leakage) from leaves of *Arabidopsis* and barley watered with NaCl or water. (D) Images of drought-stressed barley seedlings and mature plants. (E) Relative water content and photosynthesis response of barley to drought. (F) Responses of barley and *Arabidopsis* stomata to ABA application. (G) Response of *Arabidopsis* seedlings to heat stress treatments. BT, basal thermotolerance; SAT and LAT, short- and long-term acquired thermotolerance; TMHT, thermotolerance to moderately high temperature. (H) Conductivity of *Arabidopsis* leaves treated with MV. (I) Relative weight of mature *Arabidopsis* plants grown under neutral days. (J) Flowering time (days) and leaves at flowering of *Arabidopsis* plants. Error bars indicate SEM; letters one-way ANOVA; Tukey’s test. ^∗∗∗^p < 0.005; ^∗∗^p < 0.01; ^∗^p < 0.05. See also Figure S1.

Because plants are sessile organisms, they must cope with abiotic stresses through biochemical or physiological adaptations and responses. In particular, plants have evolved sophisticated molecular mechanisms to enhance survival in response to stresses, such as flooding [6], drought, salinity, and both increased and decreased temperatures [7, 8]. The Cys-Arg/N-end rule pathway controls homeostatic response of plants to low oxygen and nitric oxide (NO) through controlled degradation of group VII Ethylene Response Factor (ERFVII) transcription factors. In the presence of both gases, amino-terminal (Nt)-cysteine is oxidized, allowing arginylation by ARGINYL TRANSFERASES (ATEs) [9], which permits recognition by the N-recognin E3 ligase PROTEOLYSIS 6 (PRT6) and degradation through the 26S proteasome (Figure 1A). Absence of either gas results in substrate stabilization [3, 4]. ERFVIIs have previously been associated with plant responses to several abiotic stresses [10, 11, 12]. Other than low oxygen, no molecular mechanism has been described to explain their role, or the possible involvement of the Cys-Arg/N-end rule pathway, in physiological response to abiotic stresses. We analyzed the response of the distantly related flowering plant model genetic species *Arabidopsis thaliana* (*Arabidopsis*, a representative dicotyledon species) and *Hordeum vulgare* (barley, a representative monocotyledon species) to salinity, high temperature, drought, and oxidative stress to determine the role of the Cys-Arg/N-end rule pathway in abiotic stress responses. Initially, we studied plants lacking N-end rule E3 ligase PRT6 function; in *Arabidopsis* of the *prt6-1* null mutant; and, in barley, an *HvPRT6* RNAi line with greatly reduced expression of this gene [13, 14]. In these plants, substrates of the Cys-Arg/N-end rule pathway, including ERFVIIs, are constitutively stable [3, 14, 15]. In contrast to *Arabidopsis* wild-type (WT) and barley non-transgenic segregant (“null”) controls, plants with reduced *PRT6* function showed enhanced survival of all stresses tested. The *Arabidopsis prt6* mutant enhanced survival on high-salt media and caused hypersensitivity of germination on salt, and both *Arabidopsis prt6* and barley *HvPRT6* RNAi plants showed enhanced growth and development on soil watered with salt compared to WT and null controls (Figures 1B, S1A, and S1B). For both, cellular damage (measured as electrolyte leakage) was higher in WT/null leaves (Figure 1C). Drought stress is a major limitation to crop production, and a large body of evidence has demonstrated the role of abscisic acid (ABA) regulation of stomatal closure in the response of plants to water deficit [16]. Barley *HvPRT6* RNAi plants showed tolerance to drought treatment in comparison to null controls (Figures 1D and 1E), although we could not observe a consistent response of *Arabidopsis prt6* to drought (Figure S1C). Stomatal closure responses in both species showed hypersensitivity to ABA application in comparison to controls (Figure 1F), which for *prt6* in *Arabidopsis* required de novo protein synthesis (Figure S1D). Genetic evidence demonstrated that this ABA hypersensitivity is regulated independently of the core SnRK2-PP2C ABA transduction pathway (Figure S1E), as also shown for other mechanisms, including the PYRABACTIN RESISTANCE-LIKE 8 (PYL8) ABA receptor promotion of lateral root growth [17] and a chloroplast retrograde signaling pathway involved in stomatal closure [18].

High-temperature stress reduces the productivity and quality of crops, enhances reactive oxygen species (ROS) production, and increases damage to cellular integrity [19]. Four different thermotolerance assays were performed to assess the role of the N-end rule pathway in *Arabidopsis* in response to heat stress to evaluate basal thermotolerance (BT), short- and long-term acquired thermotolerance (SAT and LAT), and thermotolerance to moderately high temperature (TMHT), the four major thermotolerance types known in plants [20]. *prt6* showed very high BT (as did a double mutant removing ATE activity [Figure 1A], *ate1 ate2*), substantially outperforming the WT control. The *prt6* mutant also showed slightly increased SAT and LAT, demonstrating that stabilized N-end rule substrates could increase tolerance to severe high temperature (Figure 1G). In contrast, *prt6* showed substantially reduced TMHT in comparison with WT. As salinity, drought, and heat shock all cause oxidative stress, we analyzed *Arabidopsis* seedling response to methyl viologen (MV), an herbicide that induces ROS generation. The oxidative stress produced, and consequent cell damage measured as electrolyte leakage, was significantly lower in *prt6* (Figure 1H).

The five *Arabidopsis* ERFVII AP2-domain transcription factors (RELATED TO APETALA 2.12 [RAP2.12], RAP2.2, RAP2.3, HYPOXIA RESPONSIVE ERF 1 [HRE1], and HRE2) are the only known physiological substrates of the Cys/Arg-N-end rule pathway in plants [1, 2, 3]. Ectopic overexpression of ERFVII-related proteins from several species has been shown to enhance tolerance to multiple abiotic stresses, though the molecular mechanism controlling this is not known [12] (and references therein). In *Arabidopsis*, using pentuple (*rap2.12 rap2.2 rap2.3 hre1 hre2*, hereafter *erfVII*) and sextuple (*prt6 erfVII*) mutants [21], we analyzed the role of Cys-Arg/N-end-rule-pathway-mediated stabilization of ERFVIIs on stress tolerance. For all stresses tested, removal of *ERFVII* functions reduced stress tolerance in WT background and removed the enhanced stress tolerance in *prt6*. Compared to WT and *prt6*, *erfVII* and *prt6 erfVII* mutants showed, respectively, reduced tolerance to salt for growth and cellular damage (Figures 1B and 1C). Enhanced ABA sensitivity of *prt6* stomata was ERFVII dependent, and mutation of Cys-2 of the ERFVII RAP2.2 to the stabilizing residue Ala (in the construct *promRAP2.2*:*MA-RAP2.2*) was sufficient to enhance stomatal ABA sensitivity equivalent to that of *prt6* (Figure 1F). This shows that stomatal ABA response is controlled by ERFVIIs, dependent on Cys-2 regulation via the N-end rule pathway. In agreement with these observations, it was shown that rice *SUBMERGENCE 1A* (*SUB1A*), an ERFVII that is apparently divorced from N-end rule regulation [1], enhances response to drought following flooding [22]. For heat shock, increased BT, SAT, and LAT caused by *prt6* was diminished by removal of *ERFVII* function (Figure 1G), demonstrating that ERFVIIs are required for the enhanced thermotolerance shown in *prt6*. The different responses of *prt6 erfVII* and *prt6* for TMHT suggest that the N-end rule pathway plays an opposite role in response to severe and moderate heat stress. Cellular damage in response to MV was significantly greater in *prt6 erfVII* than *prt6* (Figure 1H), indicating that stabilized ERFVIIs control plant response to raised ROS, in agreement with recent data showing that ERFVIIs enhance transcription of *RESPIRATORY BURST OXIDASE HOMOLOG D* (*RbohD*) [23].

We analyzed whether the Cys-Arg/N-end rule pathway and ERFVIIs provide a function during normal plant growth and development. In rice, the *SUB1A* locus provides protection against flooding by promoting a quiescence strategy in seedlings [6], indicating that one capacity of ERFVIIs may be to retard plant growth. *Arabidopsis prt6* plants are smaller than WT, *erfVII*, and *prt6 erfVII* (Figure 1I). In addition, both *erfVII* and *prt6 erfVII* mutants flower earlier than Col-0 or *prt6*, and under short-day conditions, *prt6* took longer to flower than Col-0 (Figure 1J). These data indicate that, under normal, un-stressed conditions, stabilized ERFVIIs retard plant growth, which presumably may have an adaptive advantage.

Together, these results demonstrate that, in addition to a known function in response to hypoxia via direct O_2_ sensing, Cys-Arg/N-end rule pathway regulation of ERFVIIs has a far broader function as an important component controlling plant responses to diverse abiotic stresses.

### *NITRATE REDUCTASE* Opposes Nt-Cys-Mediated Substrate Stabilization and *ERFVII* Action

**Figure 2:**
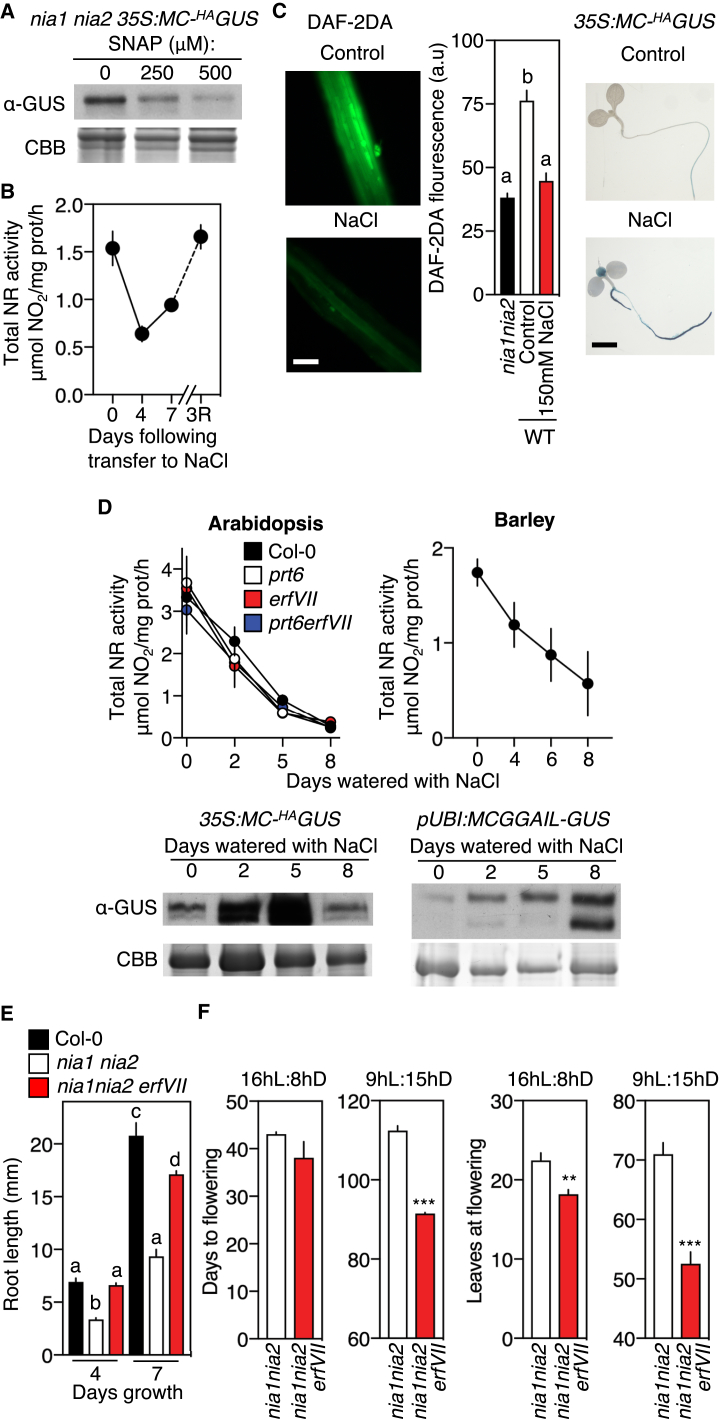
Opposing Expression and Activities of NR, an Nt-Cys Artificial Substrate, and ERFVIIs (A) Destabilization of C-^HA^GUS in *Arabidopsis nia1 nia2* by the NO donor SNAP. (B) NR activity in *Arabidopsis* 3-day-old seedlings transferred to 150 mM NaCl for 7 days and then returned to control media for 3 days (3R). (C) NO levels (measured as DAF-2DA fluorescence, arbitrary units) and histochemical visualization of C-^HA^GUS after 4 days growth on 150 mM NaCl or control media. (D) NR activity and GUS protein levels in response to watering with NaCl in *Arabidopsis* (200 mM) and barley (300 mM). (E) Root length of *Arabidopsis* seedlings at increasing time following germination. (F) Flowering time (days) and leaves at flowering of *Arabidopsis* plants. Error bars indicate SEM; letters one-way ANOVA; Tukey’s test. ^∗∗∗^p < 0.005; ^∗∗^p < 0.01. See also Figure S2.

A role for N-end-rule-regulated ERFVIIs in enhancing tolerance to abiotic stresses indicates that Nt-Cys substrates are stabilized in response to stress. This could occur through several mechanisms, including reduced O_2_ or NO levels or N-terminal shielding of substrates. Cys-Arg/N-end rule NO sensing was shown in animals to coordinate angiogenesis through the NO-sensing capacity of the substrate REGULATOR OF G-PROTEIN SIGNALING 4 (RGS4) [24]. In plants, ERFVIIs (with the conserved Met^1^-Cys^2^-amino-terminal residues) were shown to act via Nt-Cys as NO sensors through the Cys-Arg/N-end rule pathway (following Met removal by MetAP activity; Figure 1A). This mechanism regulates several developmental processes, including seed germination, stomatal closure, and hypocotyl elongation [3]. We investigated the possibility that Nt-Cys substrate stabilization in response to stress may be related to reduced NO levels. NITRATE REDUCTASE (NR) is the major source of NO in plants, and flowering plants do not contain NO synthase enzymes [5, 25, 26]. NR activity has been shown to decline strongly in response to abiotic stresses, which may result from a stress-induced decrease in the rate of photosynthetic CO_2_ assimilation to balance metabolism with growth capacity [27, 28, 29]. As the Cys-Arg/N-end rule pathway is an NO sensor [3], we hypothesized that a reduction in NR activity in response to stress may result in lowered NO levels and consequential stabilization of Cys-Arg/N-end rule substrates. To test this hypothesis, we analyzed the relationship between NR activity, NO levels, and N-end rule substrate stability and function in response to stress. We analyzed the in vivo stability of constitutively expressed artificial Cys-Arg/N-end rule activity sensors, MC-^HA^GUS (construct *35S*:*MC-*^*HA*^*GUS*) in *Arabidopsis* and MCGGAIL-GUS (construct *pUBI*:*MCGGAIL-GUS*, containing the first highly conserved seven residues of ERFVIIs) in barley [3], in relation to abiotic stress and NR activity. Due to co-translational MetAP activity (Figure 1A), Cys-2 is exposed in vivo for both proteins. The C-^HA^GUS protein is constitutively stabilized in the NR null mutant *nia1 nia2*, which has very low levels of NO [30], but destabilized in *nia1 nia2* in the presence of the NO donor S-nitroso-N-acetyl-DL-penicillamine (SNAP), confirming that C-^HA^GUS acts as an NO sensor (Figure 2A). Transfer of *35S*:*MC-*^*HA*^*GUS* WT seedlings to media plates containing increased NaCl resulted in a reduction in NR activity that was associated with a large reduction in NO levels in the roots (measured by DAF-2DA fluorescence) and increase in stability of C-^HA^GUS (Figures 2B and 2C). This indicates, as we previously showed for the ERFVII HRE2 [3], that NR-derived NO levels regulate Nt-Cys substrate stability. In *Arabidopsis* and barley, watering plants in soil with a saline solution resulted in large declines in NR activity, and drought treatment of barley also led to a big decrease in NR activity (Figures 2D and S2A). Concomitantly, the stability in leaves of C-^HA^GUS in *Arabidopsis* and CGGAIL-GUS in barley increased (Figures 2D, S2B, and S2C). We next analyzed the genetic interaction between *NR* and *ERFVII*s by comparing plant phenotypes for the NR null mutant in the presence or absence of ERFVII function (*nia1 nia2* and septuple mutant *nia1 nia2 erfVII*). *nia1nia2* plants grew much more slowly and flowered later than WT, whereas removal of *ERFVII* function greatly increased speed of growth and time to flowering (Figures 2E and 2F), demonstrating a role for NR in opposing the repressive action of ERFVIIs in the regulation of plant growth and development.

### Interactions between ERFVIIs and the SWI/SNF Chromatin-Remodeling ATPase BRAHMA Influence Plant Response to Salinity and ABA

**Figure 3:**
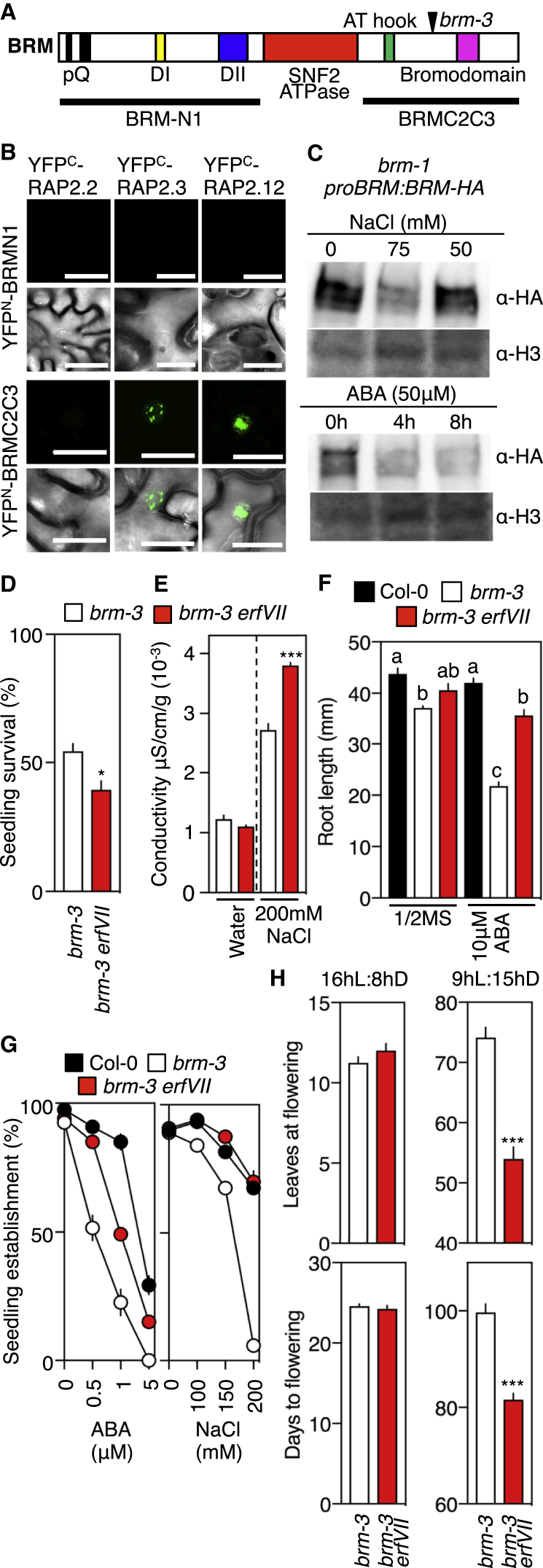
*Arabidopsis* ERFVIIs Interact Physically and Genetically with BRM (A) Diagrammatical representation of BRM showing protein domains and position of the *brm-3* mutation. (B) Laser scanning confocal imaging of *N*. *benthamiana* epidermal leaf cells infiltrated with a mixture of *A*. *tumefaciens* suspensions harboring the indicated BiFC constructs. The scale bar represents 30 μm. (C) BRM-HA protein levels in response to seedling treatment with ABA or NaCl. (D) Survival of 3-day-old *Arabidopsis* seedlings transferred to 1/2MS media containing 200 mM NaCl for 7 days followed by 5 days recovery on 1/2MS. (E) Conductivity of leaves from 24-day-old plants watered with 200 mM NaCl for 10 days. (F) Root growth following seedling transfer to media containing 10 μM ABA. (G) Establishment of 10-day-old seedlings in response to exogenous ABA or salt. (H) Flowering time (days) and leaves at flowering. Error bars indicate SEM; letters one-way ANOVA; Tukey’s test. ^∗∗∗^p < 0.005; ^∗^p < 0.05. See also Figure S3.

The SWI/SNF (switch/sucrose non-fermentable) nucleosome-remodeling complexes are important regulators of growth and development [31]. One component, the chromatin-remodeling ATPase BRAHMA (BRM) integrates plant responses to abiotic stresses, involving interactions with hormone functions that include ABA [32, 33]. Previously, we showed that ERFVIIs positively promote seed dormancy and ABA sensitivity by enhancing *ABSCISIC ACID INSENSITIVE 5* (*ABI5*) promoter activity through a double GCC *cis* element, bound by ERFVIIs, present in the ABI5 promoter [3]. We observed that this *cis* element is part of the promoter sequence reported to be targeted by BRM for *ABI5* repression (Figures 3A and S3A) [32], and ERFVIIs were shown, in a large-scale yeast 2-hybrid approach, to interact with BRM [34]. In addition, BRM has been shown to inhibit ABA sensitivity and drought responsiveness of seedlings and enhance root growth [32, 35, 36], opposite effects of ERFVIIs. We therefore probed the interaction between ERFVIIs and BRM. Bimolecular fluorescent complementation (BiFC) studies showed that the constitutively expressed (at the RNA level) ERFVIIs RAP2.12 and RAP2.3, but not RAP2.2, physically interacted with the C-terminal domain of BRM (Figures 3A and 3B). Treatment with NaCl or ABA resulted in a decline in BRM protein (Figure 3C), which may be connected to the inactivation of BRM by ABA-activated SnRK2s [33]. In comparison to *brm-3* (Figure 3A; a hypomorphic allele lacking the bromo- and DNA-binding domains [37]), *brm-3 erfVII* sextuple mutant seedlings showed decreased survival on high salt and increased seedling root tolerance to ABA; mature plants showed increased leaf cellular damage in response to salt [32] and reduced time to flowering (Figures 3D–3H). These data demonstrate a role for BRM-ERFVII interactions in controlling plant growth via opposing functionalities, perhaps in competition for the same *cis* elements. In addition to the known interaction of ERFVIIs with DELLAs [38], our data suggest that stabilized ERFVIIs contribute to multiple protein-network hubs that balance growth, development, and response to environmental stresses.

### Conclusions

In addition to the known mechanisms controlling plant responses to non-hypoxic stresses [7], our data indicate that Cys-Arg/N-end rule pathway regulation of ERFVII function is a key element of plant sensing and response to multiple abiotic stresses. A feature of the response to salt stresses studied here is a large decline in NR activity, previously attributed as a response of NR regulation to drought, mediated by reduced internal leaf CO_2_ concentration resulting from decreased leaf photosynthesis and stomatal closure [27, 28, 29]. Reduced NR activity results in reduced NO levels, suggesting that the NO-sensing function of the N-end rule pathway regulates downstream responses to enhance tolerance to salinity. One such response may include an interaction between stress-stabilized ERFVIIs and BRM, providing a link between transcription factor function and chromatin remodeling. We note also that it was recently shown that the ERFVII RAP2.3 interacts with many *Aux/*IAA promoters shown to be required for plant stress tolerance [39]. These, and other interactions, provide a conduit for transduction of stabilized ERFVII functions. Stress-induced metabolic slowdown may be indirectly sensed via the effect of declining NO levels on the stability of ERFVIIs, providing a molecular mechanism linking environmental change to stress signaling. In addition, N-end rule and NR control of ERFVII stability and function influences the speed of plant growth and flowering time. We propose that, in addition to known stress-signaling pathways, plants respond to multiple abiotic stresses, which often occur simultaneously in nature [40], via the Cys-branch of the Arg/N-end rule pathway, which is a consequence of the requirement for both O_2_ [1] and NO in the oxidation of Nt-Cys [4]. This mechanism has the capacity to integrate environment, metabolism, and response to enhance plant survival.

## STAR★Methods

### Key Resources Table

**Table:** 

REAGENT or RESOURCE	SOURCE	IDENTIFIER
**Antibodies**		

Anti-β-Glucuronidase antibody	Sigma-Aldrich	G5420; RRID: AB_477020
Anti-HA/HRP monoclonal antibody	Roche	Roche (3F10)
Anti-Histone H3 antibody	Abcam	Anti-Histone H3 antibody (ab39655); RRID: AB_732921
Secondary Antibody: (goat) anti-rabbit IgG HRP conjugate	Invitrogen	G21234; RRID: AB_2536530
Antibody detection kit: Pierce ECL Western Blotting Substrate	ThermoFisher	32106
Antibody detection kit: Amersham ECL Western Blotting Detection Kit	GE Healthcare Life Sciences	RPN2108

**Bacterial and Virus Strains**		

*Agrobacterium tumefaciens* C58C1 (pCH32 35S:p19)	Pedro Rodriguez, IBMCP, Valencia, Spain	N/A

**Chemicals, Peptides, and Recombinant Proteins**		

Murashige and Skoog (MS) medium	Sigma-Aldrich	M5524
(±)-Abscisic acid	Sigma-Aldrich	A1049
DAF-2 DA (4,5-diaminofluorescein diacetate)	Sigma-Aldrich	D2813-1MG
X-Gluc solution (5-bromo-4-chloro-3-indolyl-beta-D-glucuronic acid, cyclohexylammonium salt)	X-GLUC Direct	X-Gluc

**Experimental Models: Organisms/Strains**		

*Arabidopsis thaliana* Col-0	NASC	N1092
*Arabidopsis thaliana prt6-1*	NASC	N9873
*Arabidopsis thaliana rap2.12 rap2.2 rap2.3 hre1 hre2*	Michael Holdsworth, University of Nottingham, UK [21]	*erfVII*
*Arabidopsis thaliana prt6-1 rap2.12 rap2.2 rap2.3 hre1 hre2*	Michael Holdsworth, University of Nottingham, UK [21]	*prt6 erfVII*
*Arabidopsis thaliana brm-3*	Pedro Rodriguez, IBMCP, Valencia, Spain [32]	*brm-3*
*Arabidopsis thaliana brm-3 rap2.12 rap2.2 rap2.3 hre1 hre2*	This study	*brm erfVII*
*Arabidopsis thaliana 35S:hab(W385A) prt6-1*	This study	N/A
*Arabidopsis thaliana snrk2.2 snrk 2.3 snrk 2.6 prt6-1*	This study	N/A
*Hordeum vulgare cv Golden Promise*	Guillermina Mendiondo, University of Nottingham, UK [14]	Commercial Variety
*Hordeum vulgare cv Golden Promise HvPRT6 RNAi line 55*	Guillermina Mendiondo, University of Nottingham, UK [14]	*HvPRT6 RNAi line 55*
*brm-1 null mutant containing the promoterBRM:BRM-HA transgene*	Pedro Rodriguez, IBMCP, Valencia, Spain [33]	N/A
*Nicotana benthamiana*	Pedro Rodriguez, IBMCP, Valencia, Spain	Commercial Variety

**Oligonucleotides**		

F_RAP2.2_ATG: ATGTGTGGAGGAGCTATAATC	This study	N/A
R_RAP2.2_Stop: TCAAAAGTCTCCTTCCAGCAT	This study	N/A

**Recombinant DNA**		

pSPYNE:BRMN1	Pedro Rodriguez, IBMCP, Valencia, Spain [33]	YFP^N^-BRMN1
pYFN43:BRMC2C3	Pedro Rodriguez, IBMCP, Valencia, Spain [33]	YFP^N^-BRMC2C3
pYFC43:RAP2.12	This study	YFP^C^-RAP2.12
pYFC43:RAP2.2	This study	YFP^C^-RAP2.2
pYFC43:RAP2.3	[38]	YFP^C^-RAP2.3

**Software and Algorithms**		

ImageJ/Fiji	NIH – public domain	https://imagej.nih.gov/ij/download.html

### Contact for Reagent and Resource Sharing

Further information and requests for resources and reagents should be directed to and will be fulfilled by the Lead Contact, Michael J. Holdsworth (michael.holdsworth@nottingham.ac.uk).

### Experimental Model and Subject Details

*Arabidopsis* and barley genetic materials were described before [13, 14, 21, 32, 41, 42], except for *35S:hab(W385A) prt6-1* double, *snrk2.2 snrk 2.3 snrk 2.6 prt6-1* quadruple and *brm-3 rap2.12 rap2.2 rap2.3 hre1 hre2* (in which the functions of all Group VII ERF transcription factors are removed, shortened to *erfVII* [21]) sextuple mutant, that were generated in this study. All mutants are in the Col-0 accession (Wild-Type, WT).

### Method Details

#### Analysis of plant growth

For *Arabidopsis* germination and seedling growth assays seeds were plated on half-strength Murashige and Skoog (MS) medium (Sigma-Aldrich) supplemented with additional components as described in the text, and exposed to continuous white fluorescent light (90–100 μmol m^−2^ s^−1^) at 22°C [21]. For experiments performed in adult stage plants were grown under appropriate light/dark cycles, *Arabidopsis* plants were treated 3 weeks after germination of seeds sowed directly on soil and barley plants were staged following the decimal code until stage 29 [43]. For the analysis of *Arabidopsis* response on media supplemented with NaCl, seeds were sowed on half-strength MS and, after 3 days, were transferred to plates containing half-strength MS supplemented with 200mM NaCl for 7 days. Then seedlings were transferred back to fresh half-strength MS media plates for 5 days, after which survival was scored as growth of leaves. For cell damage analysis, plants were watered with appropriate NaCl solution twice over 10 days in the case of *Arabidopsis* and 3 times a week over 3 weeks for barley. Salt accumulation in the soil was avoided by allowing excess irrigation water to drain out of the pots. Control plants were irrigated with tap water. Photographs in Figure 1B show representative phenotypes for both *Arabidopsis* and barley after 3 weeks of treatment. Analysis of root length responses on media supplemented with ABA was carried out as previously described [32]: *Arabidopsis* seeds were sowed on half-strength MS and after 3 days transferred to plates containing half-strength MS supplemented with 10μM ABA for 7 days, after which root length was scored.

Thermotolerance assays were performed as previously described with modifications [44]: For the basal thermotolerance (BT) assay, 5 day-old seedlings were treated for 23-26 min at 44°C. For short-term acquired thermotolerance (SAT) assay, 5 day-old seedlings were acclimated for 1 hr at 37°C, recovered for 2 hr at 22°C and then treated for 170 min at 44°C. For the long-term acquired thermotolerance (LAT) assay, 5 day-old seedlings were acclimated for 1 hr at 37°C, recovered for 2 d at 22°C and then treated for 60 min at 44°C. For the tolerance against moderately high temperature (TMHT) assay, 5 day-old seedlings were treated for 8 d at 35°C (day)/33°C (night) with a 16-h day length under continuous white fluorescent light (120 μmol m^−2^ s^−1^). Following the HS treatments, plants were recovered at 22°C for indicated time before the survival rates were counted. Flowering time experiments were carried out using plants grown from seed continuously in either long days (16 hr light; 8 hr dark) or short days (9 hr light; 15 hr dark) at 22°C under white fluorescent light (120 μmol m^−2^ s^−1^). Both *Arabidopsis* and barley plants were subjected to drought stress by withholding watering for defined periods. Relative Water Content (RWC) in barley was measured as previously described [45]: Five segments of 5 cm diameter were excised from the middle of well-developed leaves of plants before and after drought treatment and their fresh weight (Wf) was recorded. The segments were placed for 24 hr in petri dishes filled with distilled water, under illumination in order to avoid loss of dry weight arising from respiration during hydration after which the turgid weight (Wt) was measured. The dry weight (Wd) of the leaf segments was measured after 24 hr at 80°C. RWC (%) was calculated as [(Wf-Wd) / (Wt-Wd)] x 100. Photosynthesis was measured using infrared gas exchange (Licor 6400XT). Total chlorophyll levels in barley leaves were determined by extraction and assay in 80% (v/v) acetone as previously described [46]: Leaf patches of known weight were ground in a pestle and mortar with 5 mL 80% (v/v) acetone. Samples were then centrifuged for 5 min at 1500xg to remove debris. Chlorophyll content was calculated as described in [46].

#### Stomatal aperture measurements

Strips were taken from the abaxial surface of *Arabidopsis* or barley leaves using curved fine forceps. Three to five leaves of each plant were used and epidermal strips floated on resting buffer (10mM MES, pH 6.2). Epidermal strips were then transferred to opening buffer (10mM MES, 50mM KCl, pH 6.2) for 2 hr, incubated in light and bubbled with CO_2_ free air, and kept at 20°C by placing Petri dishes containing the strips and opening buffer on the surface of the water in a glass tank whose temperature was maintained by a cooling coil and heater. The Petri dishes were illuminated by a light box containing fluorescent bulbs located beneath the water tank. The light box was run with a dimmer switch allowing the light intensity to be set at 300 μmol m^−2^ s^−1^. To allow the aeration of the opening buffer with CO_2_-free air, an air pump was used to force air through self-indicating soda lime, and then through a manifold which was connected to each Petri dish via rubber tubing and syringe needles. Air flow was adjusted to 100 mL min^−1^ to make sure that air perfused the opening buffer without displacing the epidermal peels from the buffer. Then strips were incubated for 2 hr in the same buffer containing 0 to 5 μM ABA (Sigm-Aldrich). The working standard of ABA (1 mg/L) was made by dilution of the stock solution for each experiment. Epidermal strips were removed and mounted on glass slides before stomatal apertures were analyzed by light microscopy as quickly as possible (i.e., within 0-5 min) and images captured using a fitted camera (Olympus Bx51 microscope with Olympus digital camera unit and graticule). The lengths and widths of apertures of 40 stomata per treatment per experiment were measured for each plant type using ImageJ software (National Institute of Health). Each experiment was repeated on three separate occasions using fresh plant samples on three consecutive days so that 120 stomatal apertures were measured per treatment or genotype. Aperture areas were calculated using the formula for an ellipse (π ½ (length) ½ (width)).

#### Measurement of ion leakage

Cell damage was determined by measuring ion leakage as described [47]: Assays were carried out using 25 day-old *Arabidopsis*, and barley plants at growth stage 23-24 [43]. In *Arabidopsis*, a disk of 0.6 cm^2^ per leaf from 24 leaves was excised using a hole punch; in barley 6 × 1cm^2^ leaf sections were used from 4 plants. Disks were rinsed briefly with water and floated on 5 mL of double distilled water for 6 hr at room temperature. In barley, the sections were placed at 80°C oven for 24 hr to calculate the dry weight. The conductivity of the water was measured using a Mettler-Toledo SevenGo conductivity meter.

#### Assay for Nitrate Reductase (NR) activity

Assay for NR activity was performed using *Arabidopsis* seedlings and *Arabidopsis* and barley leaves of 3.5 and 2.5 week-old plants, respectively as described previously [48] with modifications: Ground tissue was added to the extraction buffer (100mM HEPES (pH 7.5), 2mM EDTA, 2mM DTT and 1% (v/v) PVPP). The homogenate was centrifuged for 20 min at 30,000 g at 4°C, and the supernatant was added to the reaction buffer (100mM buffer HEPES (pH 7.5), 100mM KNO_3_, 10mM cysteine, 2mM NADH and 2mM EDTA) (to measure total enzyme activity). The reaction was performed at 25°C for 15 min and stopped by addition of zinc acetate (final concentration 30mM). The nitrite formed was determined following addition of 1% sulfanilamide in 1.5M HCl and 0.02% naphthylethylenediamine dihydrochloride (NNEDA) in 0.2M HCl, by determining absorbance at 540 nm.

#### NO detection by fluorescence microscopy

Endogenous NO levels were measured as previously described [49] by immersing seedlings in 10mM MES-KOH (pH 7) containing 10μM DAF-2 DA (4,5-diaminofluorescein diacetate, Sigma-Aldrich). Seedlings were shaken gently for 15 min in the dark, and subsequently washed for 20 min in 10mM MES-KOH (pH 7). The seedlings were visualized using a Leica DM5000B fluorescence microscope with excitation at 488nm and emission 520nm. Nitric oxide intensity was determined by selecting equal areas of the same root zone and analyzing with Fiji software [3].

#### Bimolecular fluorescence complementation (BiFC) analysis

BRM constructs (YFP^N^-BRMN1 and YFP^N^-BRMC2C3) were reported previously [33]. The RAP2.2 coding sequence was cloned into pCR8/GW/TOPO entry vector using the primers F_RAP2.2_ATG and R_RAP2.2_Stop and recombined by LR reaction into pYFC43 destination vector [50] to make pYFC43:RAP2.2 (giving protein YFP^C^-RAP2.2). RAP2.3 and RAP2.12 pCR8 constructs were published previously [38] and recombined into pYFC43 as for RAP2.2 to make pYFC43:RAP2.3 and pYFC43:RAP2.12 (giving proteins YFP^C^-RAP2.3 and YFP^C^-RAP2.12). BiFC analysis of interactions between BRM and ERFVIIs was carried out as previously described [33]: Pre-culture of a single isolated colony of *Agrobacterium tumefaciens* was grown in liquid media at 28°C for 2 days to saturate the culture. 1/100 dilution of the pre-culture was added to fresh culture media and grown overnight. Cells were harvested by centrifugation at 3000 × g for 30 min and re-suspended in infiltration solution (10 mM MES buffer pH5.6, 100 μM acetosyringone, 10 mM MgCl_2_) to an OD 600nm of 1. These cells were mixed with an equal volume of *A. tumefaciens* C58C1 (pCH32 35S:p19) so that the final optical density of *A. tumefaciens* solution was approximately 1.0. Bacteria were incubated for 3 hr at room temperature and then injected into young fully expanded leaves of 4- week-old *Nicotana benthamiana* plants. Leaves were examined 48–72 hr after infiltration using Confocal Laser Scanning Microscopy.

#### Analysis of protein abundance and GUS activity

For analysis of β-Glucuronidase (GUS) protein levels in *Arabidopsis* and barley protein extracts were prepared by grinding the tissue to a fine powder in liquid nitrogen, and extracted using buffer containing 50 mM Tris/HCl, pH 7.5, 0.1% (w/v) SDS and 1x complete protease inhibitor cocktail (Roche). Total protein content in samples was quantified by Bradford protocol against a BSA standard curve. Total protein was separated by SDS–PAGE, and transferred to nitrocellulose membrane (Immune-Blot PVDF, Biorad) by electroblotting. SeeBlue Plus2 Pre-Stained Standard Marker (Novex) was loaded as a reference for protein size. Membranes were reversibly stained with Ponceau S Red to check equal loading and protein integrity. Western blotting was carried out using a polyclonal Anti-β-Glucuronidase (GUS) antibody (Sigma) at 1/2,000 dilution. The primary antibody was detected using a secondary antibody ((goat) anti-rabbit IgG HRP conjugate, Invitrogen) at 1/10,000 dilution. Signal was detected using Pierce ECL Western Blotting Substrate (ThermoFisher).

Western blotting to assess BRM protein levels was carried out using *Arabidopsis brm-1* null mutant containing the *promoterBRM:BRM-HA* transgene, that restores BRM function [32]. Nuclear fractionation of proteins was performed as follows: Plant material was ground in liquid nitrogen and homogenized in Lysis Buffer (20 mM Tris-HCl pH 7.6, 25% Glycerol, 20 mM KCl, 2.5 mM MgCl_2_, 250 mM sucrose, 0.8 mM phenylmethylsulfonyl fluoride (PMSF), 5 mM β-mercaptoethanol, 1 tablet Protease Inhibitor Cocktail (Roche)/10mL of buffer). The lysate was filtered thought two layers of miracloth paper and was centrifuged at 12000 × g at 4°C for 20 min. The nuclear pellet was rinsed 3 times with Nuclei Resuspension Buffer (NRB, 20 mM Tris-HCl pH7.6, 25% (v/v) glycerol, 2.5 mM MgCl_2_, 0.5% Triton X-100, 0.8 mM PMSF, 5 mM β-mercaptoethanol). Nuclei were lysed with Medium Salt Buffer (MSB, 20 mM Tris-HCl pH7.6, 0.4 M NaCl, 1 mM EDTA, 5% glycerol, 0.5 mM PMSF, 0.1% Triton X-100, 1xProtease Inhibitor Cocktail) and nuclear soluble fraction was collected for analysis. Nuclear proteins were isolated following treatments and western blots were probed with either anti-HA antibody or anti-Histone H3 (Abcam) to show equal loading of wells with nuclear protein [51]. For analysis of BRM-HA and H3 proteins, after SDS-PAGE, wet transfer method was used for protein visualization. Proteins were transferred to a polyvinylidene difluoride (PVDF) membrane. Western blotting was carried out using the anti-HA/HRP antibody (1/1000) or anti-Histone H3 antibody (1/10,000 dilution). The anti-Histone H3 primary antibody was detected using a secondary antibody ((goat) anti-rabbit IgG HRP conjugate, Invitrogen) at 1/5,000 dilution. Detection was performed using the ECL western blotting chemiluminescent detection kit (GE Healthcare).

For histochemical analysis of β-Glucuronidase (GUS) enzyme activity, transgenic *Arabidopsis* and barley plant tissues were incubated in a buffer containing: Phosphate buffer (100mM) pH 7.0, Potassium Ferrycyanide (2mM), Potassium Ferrocyanide (2mM), Triton X-100 (0.1% v/v) and X-Gluc solution (5-bromo-4-chloro-3-indolyl-beta-D-glucuronic acid, cyclohexylammonium salt, X-GLUC Direct) (1mM). The samples were incubated at 37°C in the buffer not longer than 8 hr for *Arabidopsis* and 48 hr for barley.

### Quantification and Statistical Analysis

All experiment were performed at least three times. Statistical comparisons were conducted with GraphPad Prism 7.0 software. Horizontal lines represent standard error of the mean values in all graphs. For statistical comparisons we used Student’s t test, where statistically significant differences are reported as ^∗∗∗^ (p < 0.001), ^∗∗^ (p < 0.01), ^∗^ (p < 0.05), and one way Analysis of Variance (ANOVA) with Tukey’s multiple comparisons test, where significant differences (alpha < 0.05) are denoted with different letters.

## Author Contributions

M.J.H., J.V., G.M.M., P.L.R., J.E.G., Y.C., and K.S. conceived the project and designed experiments. J.V., G.M.M., M.P.-L., C.D., M.M., Y.J., Y.S., and M.J.H. performed the experiments. M.J.H., J.V., G.M.M., M.P.-L., M.M., P.L.R., J.E.G., and Y.C. analyzed the data. M.J.H. wrote the manuscript.

## Supplemental Information

Document S1. Figures S1–S3

Document S2. Article plus Supplemental Information
