# Supplementary material for: The Cys-Arg/N-End Rule Pathway Is a General Sensor of Abiotic Stress in Flowering Plants
Source: Curr Biol. 2017 Oct 23;27(20):3183–3190.e4. doi: 10.1016/j.cub.2017.09.006 (PMC5668231; doi:10.1016/j.cub.2017.09.006)
Supplement: Document S1. Figures S1–S3 [file mmc1.pdf]

Current Biology, Volume *27*

Supplemental Information

The Cys-Arg/N-End Rule Pathway Is a General Sensor of Abiotic Stress in Flowering Plants

Jorge Vicente, Guillermina M. Mendiondo, Mahsa Movahedi, Marta Peirats-Llobet, Yu-ting Juan, Yu-yen Shen, Charlene Dambire, Katherine Smart, Pedro L. Rodriguez, Yee-yung Charng, Julie E. Gray, and Michael J. Holdsworth

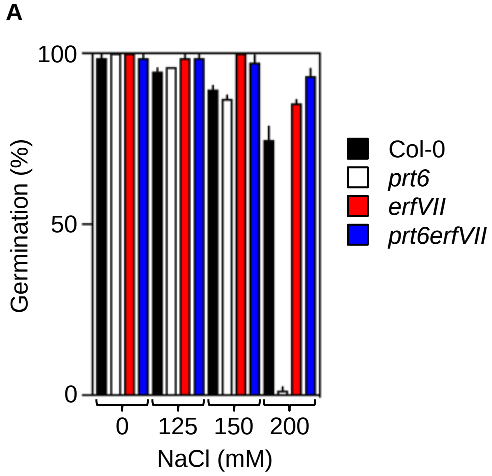

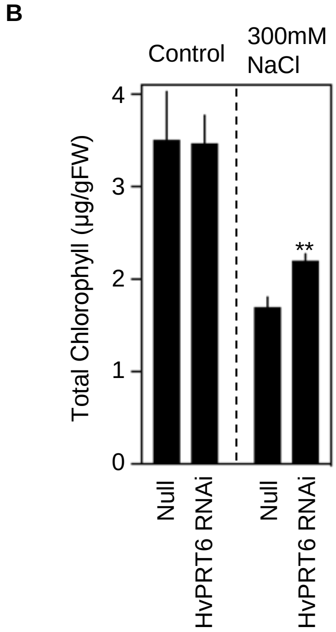

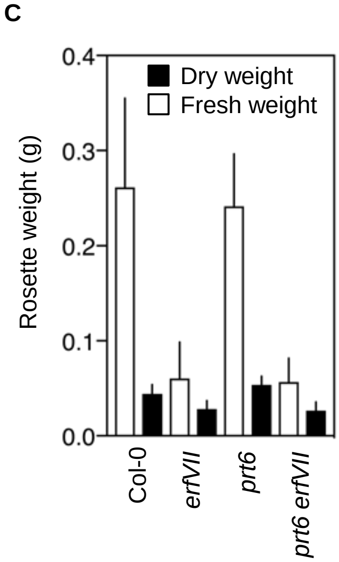

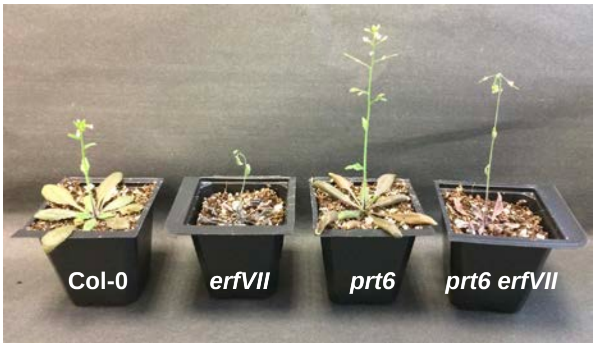

prt6 and *erfVII*- dependent response to 10 days water deprivation. Images and weights of droughted plants are shown.

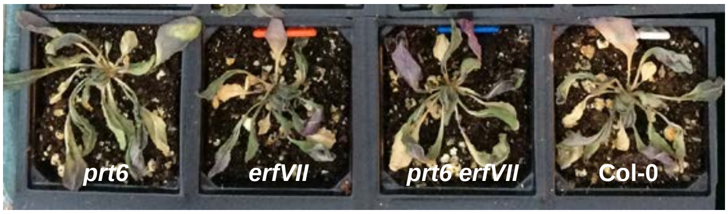

No genotype-specific response to 10 days water deprivation

D

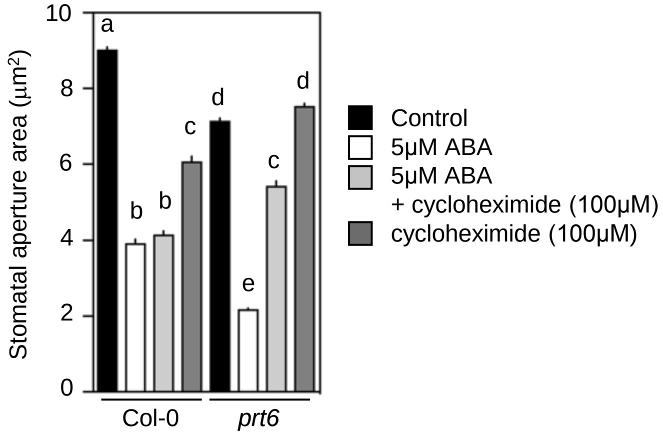

E

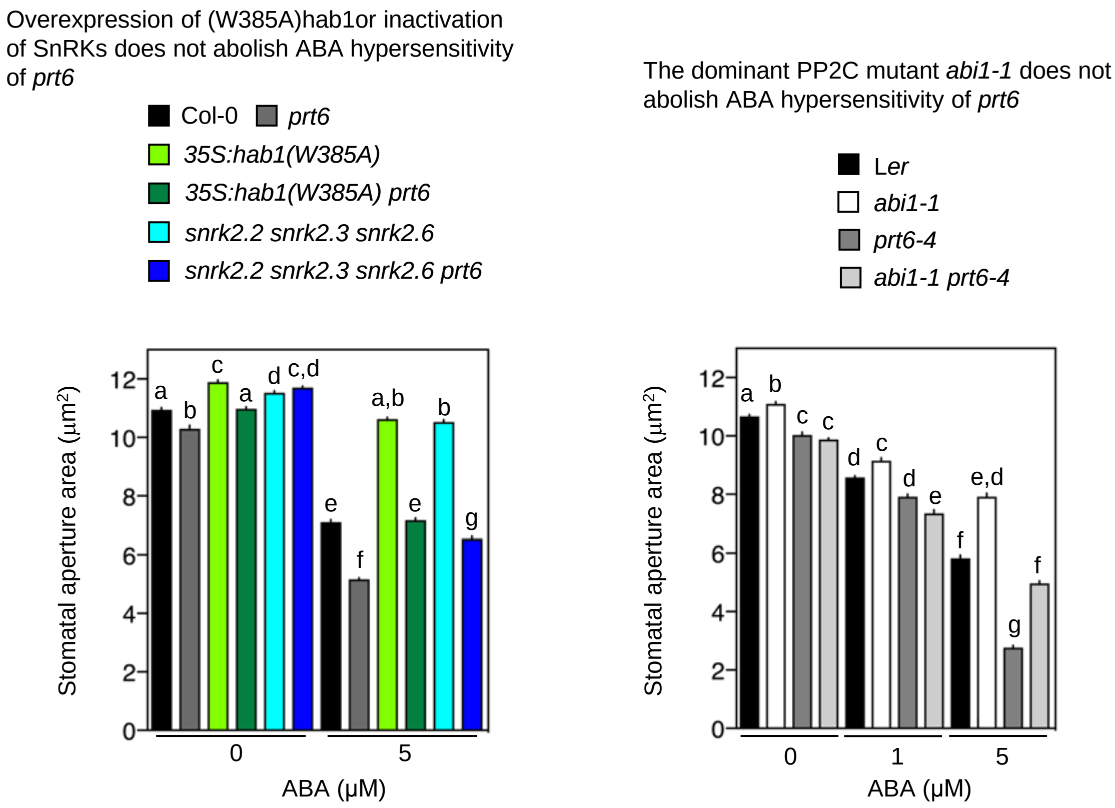

Figure S1, Related to Figure 1.
A. Sensitivity of germination to NaCl is controlled by N-end rule regulation of ERFVIIs in Arabidopsis. B. Chlorophyll content of barley plants in response to watering with 300mM NaCl. C. Inconsistent response of Arabidopsis N-end rule mutants to drought treatment. Two representative experiments are shown, where *prt6* and *erfVII*- dependent response to water deprivation were observed or not observed. The reason for this inconsistency has not been determined. **D. Stomatal ABA hypersensitivity of *prt6* requires protein synthesis. E. Stomatal ABA hypersensitivity of *prt6* is not regulated through the core PYR/PYL-PP2C-SnRK ABA transduction pathway**
Error bars indicate SEM,** = p<0.01, letters one-way ANOVA, Tukey's test.

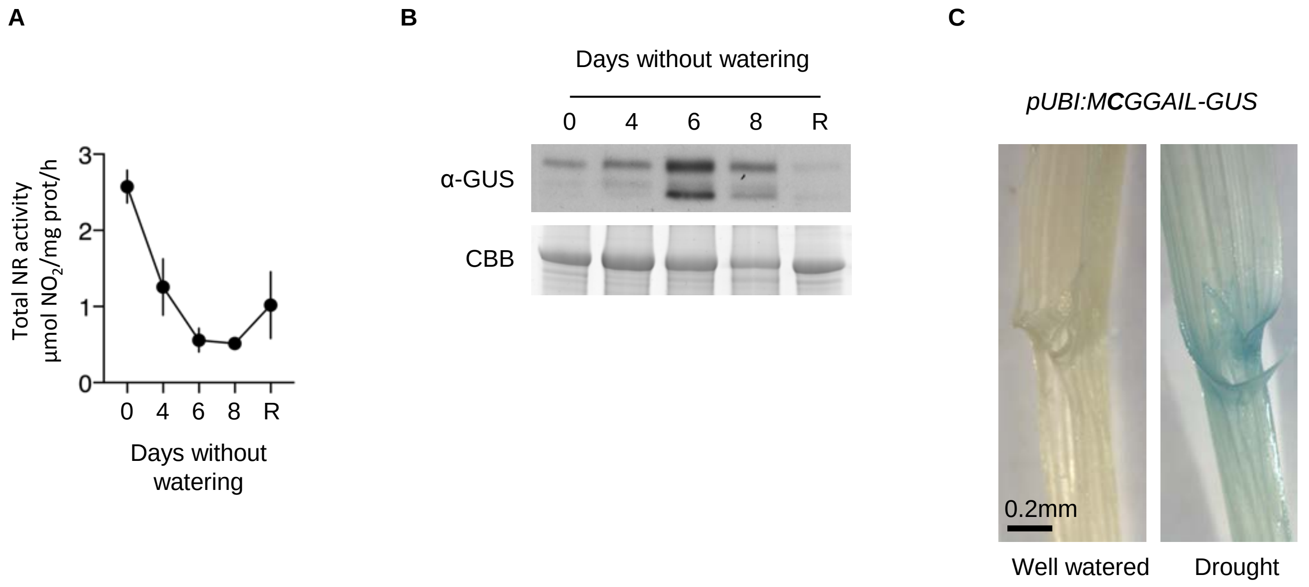

Figure S2, Related to Figure 2.
A. Nitrate reductase activity in barley in response to drought stress. R = Recovery; 3 days watered after drought. **B. Western blot analysis of MCGGAIL-GUS in barley in response to drought stress.** R = Recovery; 3 days watered after drought. **C. Histochemical analysis of MCGGAIL-GUS in barley flag leaf material in response to drought stress.** Drought = 7 days without watering. Error bars indicate SEM.

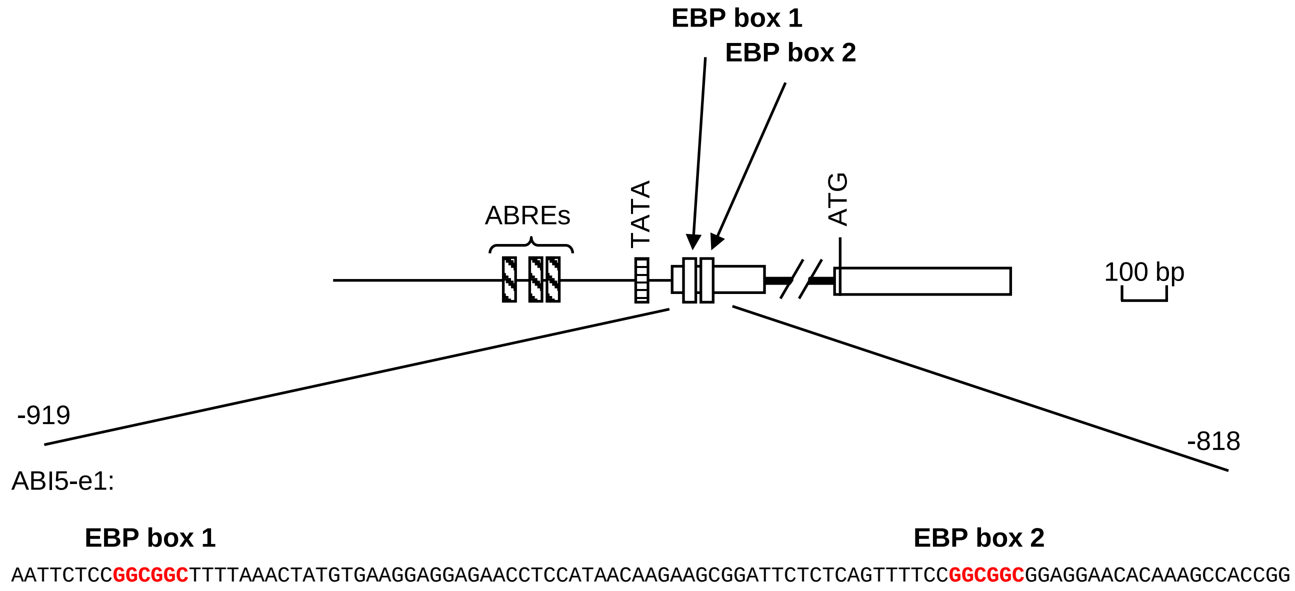

Figure S3, Related to Figure 3.
A. Overlap of BRM interaction site and ERFVII binding sites in the Arabidopsis ABI5 promoter.
ABI5-e1 taken from [S1]

Supplemental References:

S1. Han, S.K., Sang, Y., Rodrigues, A., BIO425F2010, Wu, M.F., Rodriguez, P.L., and Wagner, D. (2012). The SWI2/SNF2 Chromatin Remodeling ATPase BRAHMA Represses Abscisic Acid Responses in the Absence of the Stress Stimulus in Arabidopsis. Plant Cell 24, 4892-4906.
